# Comparison of Chemiluminescence Enzyme Immunoassay (Cl-ELISA) with Colorimetric Enzyme Immunoassay (Co-ELISA) for Imidacloprid Detection in Vegetables

**DOI:** 10.3390/foods12010196

**Published:** 2023-01-01

**Authors:** Rongqi Zhai, Ge Chen, Guangyang Liu, Xiaodong Huang, Xiaomin Xu, Lingyun Li, Yanguo Zhang, Donghui Xu, A. M. Abd El-Aty

**Affiliations:** 1 Key Laboratory of Vegetables Quality and Safety Control, Laboratory of Quality & Safety Risk Assessment for Vegetable Products, Ministry of Agriculture and Rural Affairs, Institute of Vegetables and Flowers, Chinese Academy of Agricultural Sciences, Beijing 100081, China; 2 Department of Pharmacology, Faculty of Veterinary Medicine, Cairo University, Giza 12211, Egypt; 3 Department of Medical Pharmacology, Faculty of Medicine, Atatürk University, 25240 Erzurum, Turkey

**Keywords:** imidacloprid, colorimetric assay, chemiluminescent assay, enzyme-linked immunoassay

## Abstract

Imidacloprid is one of the most commonly used insecticides for managing pests, thus, improving the quality and yield of vegetables. The abuse/misuse of imidacloprid contaminates the environment and threatens human health. To reduce the risk, a colorimetric enzyme-linked immunoassay assay (Co-ELISA) and chemiluminescence enzyme-linked immunoassay assay (Cl-ELISA) were established to detect imidacloprid residues in vegetables. The linear range of Co-ELISA ranged between 1.56 μg/L and 200 μg/L with a limit of detection (LOD) of 1.56 μg/L. The values for Cl-ELISA were 0.19 μg/L to 25 μg/L with an LOD of 0.19 μg/L, which are lower than those of Co-ELISA. Fortifying Chinese cabbage, cucumber, and zucchini with imidacloprid at 10, 50, and 100 μg/L yielded recoveries between 81.7 and 117.6% for Co-ELISA and at 5, 10, and 20 µg/L yielded recoveries range from 69.7 to 120.6% for Cl-ELISA. These results indicate that Cl-ELISA has a high sensitivity and a rapid detection time, saving cost (antigen and antibody concentrations) and serving as a more efficient model for the rapid detection of imidacloprid residue.

## 1. Introduction

Imidacloprid (IMI), a neonicotinoid insecticide, is widely used for pest control in agriculture, ensuring the yield and quality of vegetables [1]. Although IMI is highly efficient for pest control, its misuse and abuse are expected to seriously threaten the ecosystem and public health [2,3]. IMI fails to degrade completely, resulting in long persistence [4]. Long-term exposure to IMI causes neurological damage, which poses a great risk to human health [5,6]. Hence, a sensitive analysis of IMI residues in foods is required to reduce health and environmental risks from hazardous materials.

Many instrumental methods, such as liquid chromatography (LC) [7], liquid chromatography–tandem mass spectrometry (LC—MS/MS) [8], and gas chromatography–tandem mass spectrometry (GC—MS/MS) [9], have been used to determine IMI residues. However, these methods are costly, complex, and time-consuming [10]. Rapid detection methods (such as immunoassays [11,12], aptamer methods [13], and electrochemical sensor methods [14,15]) with high sensitivity, simple operation, and low cost have been developed to overcome the shortcomings of these methods. In immunoassays, antibodies have the property of highly sensitive molecular recognition [16]. Enzyme-linked immunoassay (ELISA), as one of the immunoassays for the rapid evaluation of neonicotinoid insecticides, consists of two main formats: direct competitive ELISA (DC-ELISA) and indirect competitive ELISA (IC-ELISA) [17,18,19]. IC-ELISA is widely used to detect pesticide residues because of its advantages, such as high sensitivity, simplicity, simple pretreatment, and high throughput detection. For instance, Zhang et al. [20] used the colorimetric (Co) IC-ELISA method to determine IMI residues with an LOD of 0.025 mg/L, showing high sensitivity and stability. Yue et al. [21] also used Co-IC-ELISA to determine organophosphate pesticides in agricultural products, obtaining an LOD for methyl parathion of 1.94 ng/mL. The method yielded favorable recoveries of 84.16–106.96% at a low spiking level. Compared to colorimetric assays, chemiluminescence, as a highly sensitive detection method, is also used to rapidly detect IMI. For instance, Girotti et al. [22] used chemiluminescence to detect IMI in honey using IC-ELISA. They found that the LOD was 0.11 ng/mL. Similarly, Hu et al. [23] detected IMI based on IC-ELISA using chemiluminescence and obtained a low LOD of 0.637 ng/mL. Colorimetric and chemiluminescent assays based on IC-ELISA have been popularly used to detect IMI with low LOD. However, the LOD of the related IC-ELISA method can be further improved.

**Figure 1:**
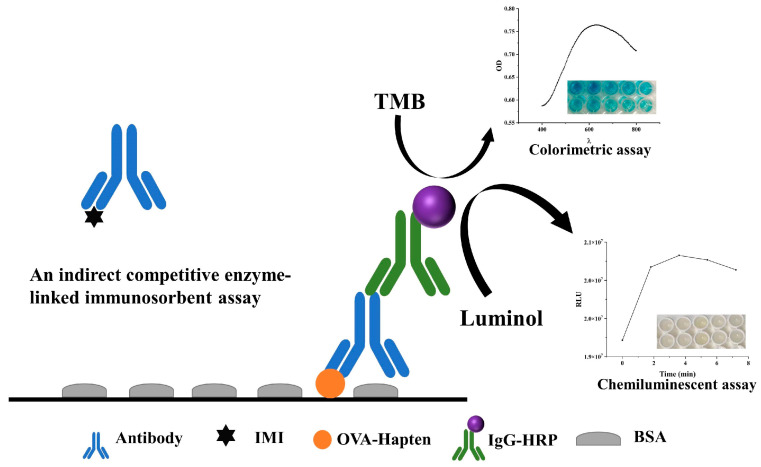
Illustration of the principle of Co-ELISA and Cl-ELISA.

To improve the LOD of IC-ELISA, this study reports colorimetric and chemiluminescent IC-ELISAs for detecting IMI (Figure 1). Comparing the sensitivity, linear range, antigen–antibody ratio, and detection time of Cl-ELISA and Co-ELISA provided an approach for selecting a detection assay. Finally, different sample preparations were developed for Co-ELISA and Cl-ELISA, which further improved the sensitivity and recovery of Co-ELISA and Cl-ELISA in vegetable samples, providing a reference for applying ELISAs.

## 2. Materials and Methods

### 2.1. Chemicals and Reagents

Imidacloprid monoclonal antibody and ovalbumin-coated hapten (OVA-Hapten) were provided by Zhejiang University (Hangzhou, China). Peroxidase-conjugated AffiniPure goat antimouse IgG was purchased from Jackson ImmunoResearch Laboratories Inc. (West Grove, PA, USA). Imidacloprid standard (100 mg/kg) was procured from Beijing Manhage Bio-Technology Co., Ltd. (Beijing, China). Acetonitrile (chromatographic grade) was procured from Merck AG (Darmstadt, Germany). TMB (3,3′,5,5′-Tetramethylbenzidine) single-component substrate solution, phosphate buffer saline tablets, and tris (hydroxymethyl) aminomethane were secured from Beijing Solarbio Science & Technology Co., Ltd. (Beijing, China). HRP-chemiluminescent reagents A and B were supplied by Beijing Keyuebio Technology Co., Ltd. (Beijing, China). Albumin (bovine serum) was obtained from Shanghai Yuanye Biotechnology Co., Ltd. (Shanghai, China). Tween 20 was acquired from Shanghai Macklin Biochemical Co., Ltd. (Shanghai, China). A 96-well opaque assay plate was obtained from Shanghai Jing An Biological Technology Co., Ltd. (Shanghai, China). An immuno clear flat-bottom 96-well plate (item #439454#) was supplied by Thermo Fisher Scientific (Pittsburgh, PA, USA).

### 2.2. Optimization Parameters of the Assays

The experimental parameters (concentrations of antibody and antigen, concentration of BSA, organic solvent, and reaction time of chemiluminescence) were evaluated to improve the sensitivities of the Cl-ELISA. Seven different combinations of antigen–antibody concentrations were screened (Table 1), and the corresponding IMI calibration curves were established to obtain the best combination of antigen–antibody concentrations. When the RLU_max_/IC_50_ was greater than 5 × 10^5^, the minimum amount of antigen–antibody was desirable. The Cl-ELISA detected IMI under various concentrations of BSA (0.5, 1, 2, 3, and 5%), implementing methanol to the final volume (1, 5, 10, 20, and 30%) in the IMI dilution, and chemiluminescence reaction times (from 0 to 20 min). The negative well RLU value, positive well RLU value, and N/P value (negative well RLU value/positive well RLU value) were used as evaluation criteria in the optimization scheme to select the best physicochemical parameters. For Co-ELISA, the optimal parameters of the Co-ELISA protocol were screened according to the chessboard titrations, including OD = 1 and cost savings [24]. The optimal combination was selected by comparing 32 combinations of antigen–antibody concentrations (Table 2).

### 2.3. Sample Preparation

Method 1: Chinese cabbage, cucumber, and zucchini (purchased from local supermarkets) were selected as spiked samples. IMI standard concentrations (10, 50, and 100 μg/L) were used as fortification levels in homogenized samples (5 g) in 10 mL centrifuge tubes. The mixtures were stirred with 5 mL of methanol with manual shaking for 10 s and then left to extract for 30 min, followed by centrifugation at 8824 × *g* (4 °C) for 15 min. Afterwards, 3 mL of supernatant was transferred to a 10 mL plastic centrifuge tube. Finally, 100 µL of supernatant was transferred to a 2 mL plastic tube, and 900 µL of PBS buffer solution (10 mM, pH = 4) was added to ensure a 10% ratio of methanol.

Method 2: Chinese cabbage, cucumber, and zucchini squash (purchased from local supermarkets) were selected as spiked samples. IMI standard concentrations (5, 10, and 20 μg/L) were used as spiking levels for homogenized samples (5 g) in 10 mL centrifuge tubes. The mixtures were stirred with 5 mL of methanol with manual shaking for 10 s and then left to extract for 30 min, followed by centrifugation at 8824 × *g* (4 °C) for 15 min. The entire supernatant was transferred to a 15 mL centrifuge tube, and water was added to adjust the volume to 10 mL. Finally, 200 µL of the supernatant was transferred to a 2 mL plastic tube, and 800 µL of PBS buffer solution (10 mM, pH = 4) was added to ensure a 10% ratio of methanol.

### 2.4. Co-ELISA Establishment

A 100 μL/well OVA-Hapten (10 mg/L) in PBS (10 mM, pH 7.4) was coated on an immuno clear flat-bottom 96-well plate for 2 h. The plates were then washed three times using PBST, followed by adding a BSA (300 μL/well) blocker for 1 h. After washing three times with PBST, IMI (50 μL/well) dissolved in 10% MeOH−PBS (10 mM, pH 7.4) and antibody (2 mg/L, 50 μL/well) diluted with tris (50 mM, pH 7.4) were added and incubated for 2 h on a blocked plate. After washing, 100 μL/well of diluted (1/1000) goat antimouse IgG-HRP was added. The mixtures were incubated for 1 h, followed by the addition of TMB (100 μL/well). After incubation for 10 min, the absorbance (A_650nm_) was read with a multifunctional microplate reader (Tecan, Salzburg, Austria). All incubations were carried out on a thermomixer (Eppendorf, Hamburg, Germany) at 37 °C and 400 rpm, followed by washing three times with PBST (10 mM PBS containing 0.1% Tween 20, pH 7.4) using a DEM-3 washing machine (Tuopu, Beijing, China).

### 2.5. Cl-ELISA Establishment

A 100 μL/well OVA-Hapten (5 mg/L) in PBS (10 mM, pH 7.4) was coated on 96-well opaque assay plates for 2 h. The plates were then washed, followed by the addition and incubation of BSA (300 μL/well) blocker for 1 h. After washing, analyte (50 μL/well) dissolved in 10% MeOH−PBS (10 mM, pH 7.4) and antibody (0.5 mg/L, 50 μL/well) diluted with tris (50 mM, pH 7.4) were added and incubated for 2 h on a blocked plate. After washing 3 times, 100 μL/well of diluted (1/1000) goat antimouse IgG-HRP was added. The mixtures were incubated for 1 h, followed by the addition of HRP-chemiluminescent reagents A and B (50 μL/well). After incubation for 3.6 min, the luminescence (total luminescence) was read with a multifunctional microplate reader (Tecan, Salzburg, Austria). All incubations were carried out on a thermomixer (Eppendorf, Hamburg, Germany) at 37 °C and 400 rpm, followed by washing three times with PBST (10 mM PBS containing 0.1% Tween 20, pH 7.4) using a DEM-3 washing machine (Tuopu, Beijing, China).

### 2.6. Calibration Curves

The calibration curve for Cl-ELISA was plotted by considering IMI concentrations on the x-axis against percent inhibition on the y-axis. The percent inhibition was calculated using the following Equation (1) [25,26]. Similarly, the Co-ELISA was calculated using Equation (2).

(1) $$I\%\;=\;\left(1-\frac{{\mathrm{RLU}}_{x}{-\mathrm{RLU}}_{\min}}{{\mathrm{RLU}}_{\max}{-\mathrm{RLU}}_{\min}}\right)\;\times \;100\%$$

(2) $$I\%\;=\;\left(1-\frac{{\mathrm{OD}}_{x}{-\mathrm{OD}}_{\min}}{{\mathrm{OD}}_{\max}{-\mathrm{OD}}_{\min}}\right)\;\times \;100\%$$

In Equation (1), RLU_max_ and RLU_x_ are the response without IMI and the response value when the concentration of the standard solution is x, respectively. RLU_min_ is the response value of the blank control.

In Equation (2), OD_max_ and OD_x_ are the response without IMI and the response value when the concentration of the standard solution is x, respectively. OD_min_ is the response value of the blank control.

## 3. Results

### 3.1. Optimization Parameters of the Assays

#### 3.1.1. Antigen and Antibody Concentrations

The parameters of the Cl-ELISA protocol were optimized according to the chessboard assay, including signal values greater than 5.0 × 10^5^, high RLU_max_/IC_50_, significant pesticide inhibition effects, and cost savings [24,26]. For Cl-ELISA, the maximum value of RLU_max_/IC_50_ means a larger detection range and the highest sensitivity for chemiluminescence [27]. As shown in Table 1, a combination of 5 mg/L coated antigen and 0.5 mg/L antibodies were selected in the Cl-ELISA system because R^2^ = 0.996, RLU_max_ 6.24 × 10^6^ was greater than 5.0 × 10^5^, and cost savings. As shown in Table 1, antibody concentrations that are too high or too low at constant OVA-Hapten concentrations can decrease RLU and lower sensitivity, possibly due to the hook effect [28].

For Co-ELISA, we used a chessboard assay to screen for a combination of antigen–antibody concentrations. OD = 1 is used as a target because it can save the amount of antigen and antibody used and meet the method’s sensitivity [29,30]. A combination with OD = 1 and the smallest antigen–antibody concentration was used as the optimized concentration. As shown in Table 2, the higher the antigen and antibody concentrations were, the greater the OD value. When the antigen concentration was constant, the OD value decreased with increasing antibody dilution (antibody dilutions from 2 to 16 times); the OD value changed without an obvious pattern with increasing antibody dilution (antibody dilutions from 32 to 128 times). Because the concentration of antibodies is too low, the binding rate of antibodies and antigens may be reduced. The combinations of antigens with an antibody with OD values of approximately 1.0 were 20 mg/L with 4 mg/L and 10 mg/L with 2 mg/L, and the combination of 10 mg/L with 2 mg/L was chosen according to cost savings.

#### 3.1.2. Blocking Agent

**Figure 2:**
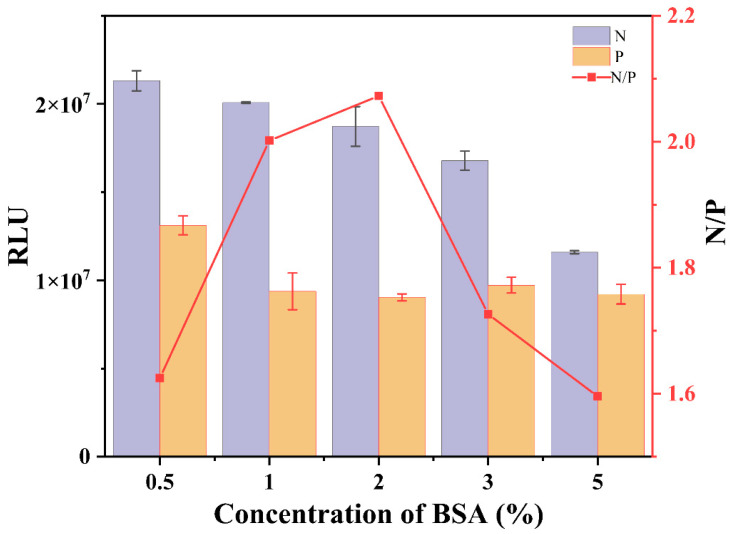
Effects of BSA on Cl-ELISA. The data are averages of three replicates. P: the RLU values of the positive wells (the concentration of IMI = 3.125 μg/L), N: the RLU values of the negative wells (the concentration of IMI = 0).

As a critical step, protein antibodies or antigens are immobilized on plastic surfaces through nonspecific binding in ELISA. Binding nonspecific proteins may lead to a decline in sensitivity and specificity and can also produce false negative results [25]. To avoid nonspecific binding, proteins were used to block the vacant sites in the plastic well. As one of the most commonly used blocking agents, BSA effectively showed optimum results in ELISA [31,32]. Therefore, BSA was chosen as the blocking solution and tested in the 0.5–5% range. According to the results (Figure 2), with increasing BSA concentration, the RLU values of the negative wells decreased continuously. The results showed that as the concentration of BSA increased, the excessive BSA adsorption on the plastic plates hindered the binding of antigens and antibodies. With increasing BSA concentration, as shown in Figure 2, the RLU values of the positive wells decreased and remained unchanged. The results suggest that the low concentration of BSA could not wholly block the adsorption sites of the plastic plates, which quickly produced false negatives. When the N/P value is the maximum, its chemiluminescence value is larger, and the sensitivity is higher. A concentration of 2% BSA was chosen as the blocking concentration.

#### 3.1.3. Methanol

**Figure 3:**
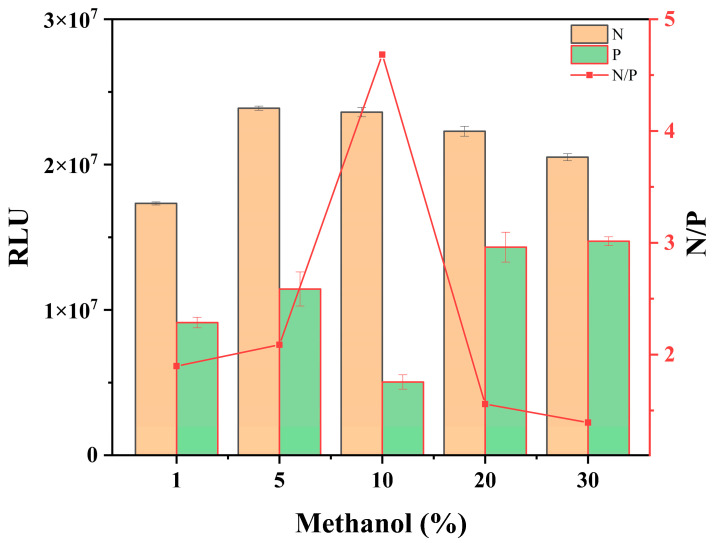
Effects of methanol on Cl-ELISA. The data are averages of three replicates. P: the RLU values of the positive wells (the concentration of IMI = 3.125 μg/L), N: the RLU values of the negative wells (the concentration of IMI = 0).

IMI solubility in water is less than that in organic solvents [33], and adding an appropriate amount of organic solvent to the buffer solution assists in the dissolution of IMI. However, an excessively high solvent content might affect the antigen–antibody reaction [34]. To compare the effect of different methanol contents on Cl-ELISA, PBS buffers containing 1%, 5%, 10%, 20%, and 30% volumes of methanol were prepared to dilute the standard solutions. The results (Figure 3) show that the RLU of the negative wells decreased with increasing methanol volume from 5% to 30%. This indicates that a high methanol volume affects antigen–antibody reactions. When the volume of methanol was 10%, the chemical RLU of the positive wells was the smallest, and the value of N/P was the largest at 4.6. The PBS buffer containing 10% methanol was selected for the Cl-ELISA method to prepare the standard pesticide solution under the conditions of pesticide solubility, maximum signal value, and detection sensitivity.

#### 3.1.4. Reaction Time

**Figure 4:**
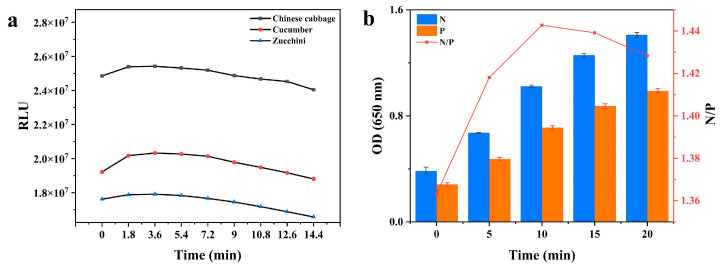
(**a**) The dynamic curve of Cl-ELISA in the three tested vegetable samples from 0 to 14.4 min with no analyte. (**b**) Absorbance values for optimization of reaction time (0, 5, 10, 15, and 20 min) for Co-ELISA.

The substrate action time is an important factor affecting the sensitivity of Cl-ELISA. A reaction time that is too short or too long might result in a false negative. The chemiluminescence dynamic curves of the 0-well plates were analyzed under three different matrices to control the reaction time. The results (Figure 4a) showed that the RLU tended to increase and decrease with time. The plateau stabilization period of chemiluminescence (1.8–5.4 min) was approximately 3.6 min. At 3.6 min, the chemiluminescence values reached a maximum in the three matrices: cabbage 2.5 × 10^7^, cucumber 2.0 × 10^7^, and zucchini 1.7 × 10^7^.

Under optimal conditions, Co-ELISA was set at 37 °C with substrate action times of 0, 5, 10, 15, and 20 min (Figure 4b). With increasing time, the OD values of the negative and positive wells increased. At 10 min, the OD value was approximately 1.0, and the N/P value was a maximum. Thus, 10 min was chosen as the best action time for the TMB solution. The time of the maximum N/P value was chosen as the optimal action time (10 min) of the TMB solution. The RLU of Cl-ELISA could be measured immediately after the addition of the HRP-chemiluminescent reagents (3.6 min), while the colorimetric method required 10 min of reaction.

### 3.2. Calibration Curves

**Figure 5:**
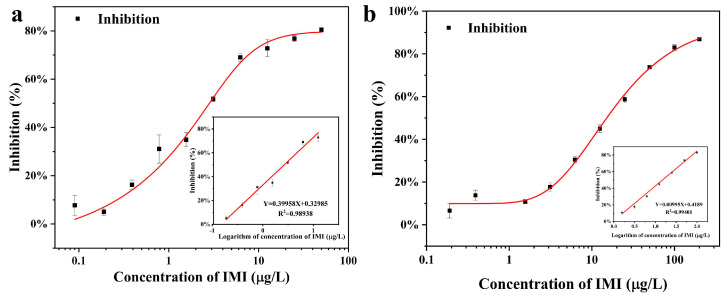
(**a**) Standard curve for the detection of IMI using Cl-ELISA. (**b**) Standard curve for the detection of IMI using Co-ELISA.

All these factors were accounted for, and the optimal conditions for Cl-ELISA were as follows: coated antigen (5 mg/L) and antibody (0.5 mg/L) produced the highest RLU_max_/IC_50_ ratio; 10% methanol, 2% BSA, and 3 min substrate action time were used for Cl-ELISA. Under optimal conditions, Figure 5a shows the Cl-ELISA standard curve for IMI. A calibration curve was obtained based on a similar linear section of the standard curve (inset of Figure 5a). The limit of detection (LOD) and the sensitivity (IC_50_) of Cl-ELISA were 0.19 μg/L and 2.66 μg/L, respectively. Similarly, the optimal conditions for Co-ELISA were as follows: coated antigen (10 mg/L) and antibody (2 mg/L) produced the OD = 1; 10% methanol, 2% BSA, and 10 min substrate action time were used for Co-ELISA. Under optimal conditions, Figure 5b shows the Co-ELISA standard curve for IMI. A calibration curve was obtained based on a similar linear section of the standard curve (inset of Figure 5b). The limit of detection (LOD) and the sensitivity (IC_50_) of Cl-ELISA were 1.56 μg/L and 8.15 μg/L, respectively.

Figure 5 shows the Co-ELISA linearity over a wide range (1.56–200 μg/L) with R^2^ = 0.9893. In comparison, the Cl-ELISA linearity range (0.19–25 μg/L) with R^2^ = 0.9940 may be caused by the concentration of antigen and antibody of Co-ELISA being greater than that of Cl-ELISA. The sensitivity of Cl-ELISA (IC_50_ = 1.56 μg/L) was greater than that of Co-ELISA (IC_50_ = 8.15 μg/L), which may be due to the sensitivity of the chemiluminescence assay compared to that of the colorimetric assay (Table 3). From Table 3, for Co-ELISA, the linearity (1.56–200 μg/L) was broader than that of Cl-ELISA (0.19–25 μg/L), which achieved quantitative analysis in two orders of magnitude ranges. For Cl-ELISA, the concentration of antigen and antibody was economical compared to Co-ELISA. The IC_50_ (1.56 μg/L) of Cl-ELISA was more sensitive than the IC_50_ (8.15 μg/L) of Co-ELISA, which was caused by the chemiluminescence assay being more sensitive than the colorimetric assay. In addition, chemiluminescent assays were time-saving and efficient compared to colorimetric assays.

### 3.3. Optimization of Sample Preparation

Method 1 was the sample preparation for Cl-ELISA and Co-ELISA. Co-ELISA showed good recovery, while Cl-ELISA had poor recovery (26.8–237.5%) and RSD (1.4–52.7%) (Table 4). The recovery and RSD of Cl-ELISA could not meet the recovery (between 60 and 120%) and RSD (≤30%), according to NY/T 788-2018 [35]. Due to the mutual solubility of methanol and water, the proportion of methanol in the extracted supernatant could not be determined, thereby, reducing the sensitive detection of trace IMI in Cl-ELISA. Thus, method 1 was optimized by transferring all the supernatant to a centrifuge tube, after which water was added to fix the volume of methanol to 10 mL to ensure a constant volume of methanol. A trace amount of IMI could be extracted completely, making Cl-ELISA obtain good recovery.

### 3.4. Accuracy and Precision

**Figure 6:**
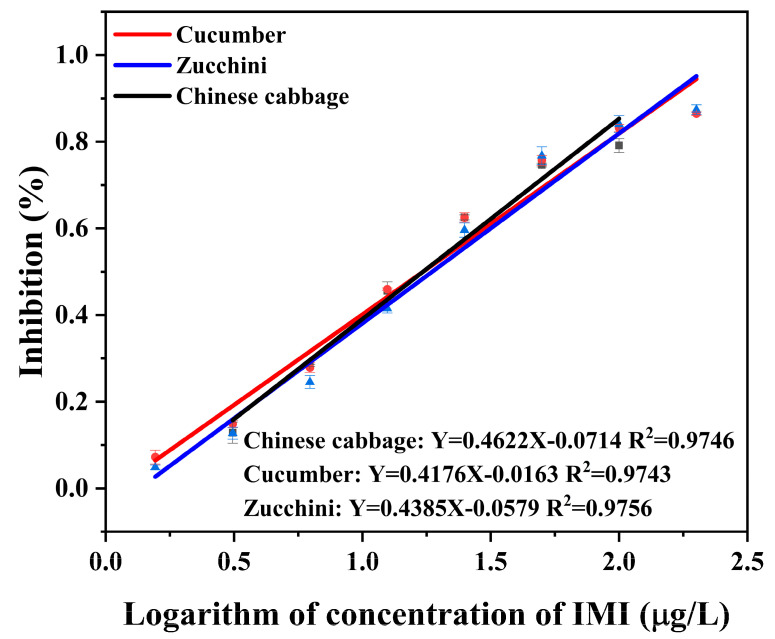
Standard curve for the detection of IMI in vegetable samples (Chinese cabbage, cucumber, and zucchini) using Co-ELISA.

**Figure 7:**
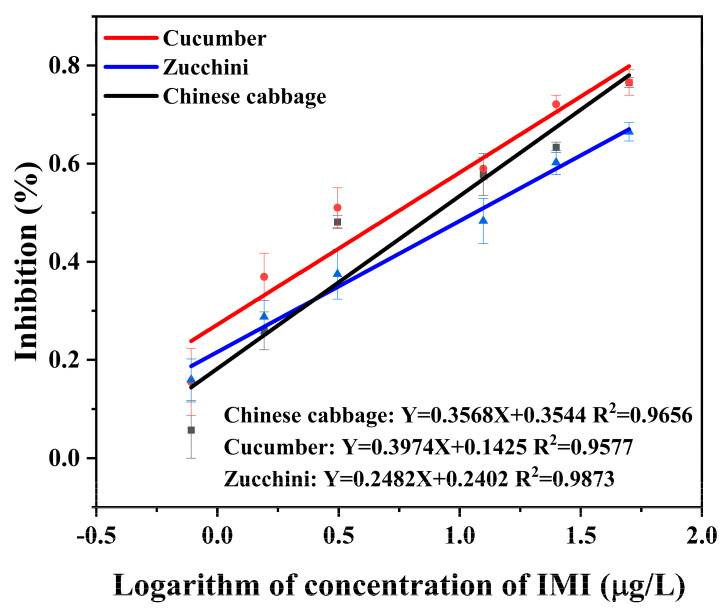
Standard curve for the detection of IMI in vegetable samples (Chinese cabbage, cucumber, and zucchini) using Cl-ELISA.

The inhibition curves for IMI in Chinese cabbage, cucumber, and zucchini matrices using Co-ELISA and Cl-ELSIA are shown in Figure 6 and Figure 7. Both Co-ELISA and Cl-ELISA achieved quantitative analysis of IMI with high sensitivity. The linearity of Co-ELISA was observed in the range of 3.125 μg/L–100 μg/L in Chinese cabbage (R^2^ = 0.9746), 1.56 μg/L–200 μg/L in cucumber (R^2^ = 0.9743), and 1.56 μg/L–200 μg/L in zucchini (R^2^ = 0.9756). The linearity of Cl-ELISA was observed in the range of 0.39 μg/L–25 μg/L in Chinese cabbage with a good regression coefficient (R^2^) of 0.9656, 1.56 μg/L–50 μg/L in cucumber (R^2^ = 0.9577), and 0.39 μg/L–50 μg/L in zucchini (R^2^ = 0.9873). Chinese cabbage, cucumber, and zucchini were spiked at concentrations of 10, 50, and 100 μg/L for Co-ELISA and concentrations of 5, 10, and 20 μg/L for Cl-ELISA. The recovery was performed under optimized conditions and compared with Cl-ELISA for its performance. According to Table 4, for Co-ELISA, the recovery of IMI in spiked samples varied from 81.7 to 117.5% in Chinese cabbage (RSD between 5.4 and 6.9%), 79.4 to 98.2% in cucumber (RSD between 0.7 and 3.2%), and 82.5 to 117.6% in zucchini (RSD between 7.9 and 11.6%). For Cl-ELISA, recovery varied between 69.7 and 109.3% in Chinese cabbage (RSD between 1.5 and 8.1%), 74.1 and 114.1% in cucumber (RSD between 1.0 and 7.9%), and 69.9 and 120.6% in zucchini (RSD between 5.0 and 15.0%). Comparing the two assays, Co-ELISA showed the best recovery results, where 79.4 to 117.6% was achieved for all samples (Table 4). At a lower concentration of 5 μg/L, Cl-ELISA showed better recovery in cucumber than in Chinese cabbage and zucchini, which could be due to matrix effects. According to China’s and the EU’s pesticide residue limit standards, (the MRL of IMI in Chinese cabbage is 0.2 mg/kg and that of cucumber and zucchini is 1 mg/kg, China) (the MRL of IMI in Chinese cabbage is 0.01 mg/kg and that of cucumber is 0.5 mg/kg, EU) Co-ELISA and Cl-ELISA can meet the requirements of rapidly detecting IMI. Co-ELISA and Cl-ELISA are suitable to ensure rapid, reliable, and sensitive detection of IMI in Chinese cabbage, cucumber, and zucchini samples.

### 3.5. Comparison with Other Methods for Detecting IMI

Table 5 shows the LOD and linear range of different instruments for detecting IMI. The LOD = 1.56 μg/L of Co-ELISA and the LOD = 0.19 μg/L of Cl-ELISA were lower than those of HPLC and HPLC—MS/MS in Table 5, which proved that Co-ELISA and Cl-ELISA were feasible. Some immunoassay methods detect IMI with significantly lower LODs than this work and show better results. For example, Wang et al. [36] used an immunochromatographic method based on scandium-tetrakis (4-carboxyphenyl) porphyrin (TCPP) metal–organic framework nanocubes to detect IMI in vegetables, and the Sc-TCPP 3D MOF had good biocompatibility and optical properties, thus, enhancing the sensitivity of the detection. In addition, Fernández et al. [37] constructed an immunosensor for the detection of IMI based on nanogold electrodes and obtained a low LOD of 22 pmol/L. Guo et al. [38] established immunoassay based on graphene oxide (GO) and up-converting nanoparticles (UCNPs) showed a wide detection range of 0.08–50 ng/mL to IMI. The combination of immunoassays and nanomaterials allows for more sensitive detection.

## 4. Conclusions

Herein, Co-ELISA and Cl-ELISA were developed for detecting IMI in Chinese cabbage, cucumber, and zucchini. The recoveries ranged from 81.7 to 117.6% for Co-ELISA and 69.7 to 120.6% for Cl-ELISA. The principle of detection used for both assays can also be applied to detect IMI at a sensitive level in other vegetable samples. Co-ELISA exhibited a wide linear range (1.56–200 μg/L) suitable for IMI testing at the microgram level. Compared with Co-ELISA, Cl-ELISA showed higher sensitivity (IC_50_ = 2.66 μg/L), benefiting the trace detection level, which was one order of magnitude higher than that of Co-ELISA. Cl-ELISA is a cost-saving alternative to Co-ELSIA in terms of the amount of antigen and antibody used and detection time. This study builds colorimetric and chemiluminescent assays based on ELISA detection of IMI and provides the theoretical basis for selecting an optical detection assay.

## Figures and Tables

**Table 1:** The optimal concentrations of antigen and antibody for Cl-ELISA.

Antibody Concentration (mg/L)	OVA-Hapten Concentration (mg/L)			
	10		5	
	RLU_max_/IC_50_	R^2^	RLU_max_/IC_50_	R^2^
4	2.15 × 10^6^	0.9676	-	-
2	7.56 × 10^5^	0.9491	-	-
1	8.47 × 10^6^	0.9266	1.49 × 10^6^	0.9307
0.5	4.19 × 10^6^	0.9582	6.24 × 10^6^	0.9960
0.1	-	-	2.31 × 10^6^	0.8743

**Table 2:** The optimal concentrations of antigen and antibody for Co-ELISA.

Antibody Dilution Times	OVA-Hapten Concentration (mg/L)			
	20	10	5	2.5
2	1.8459	1.1196	0.7011	0.5497
4	1.1879	0.8834	0.6110	0.5007
8	0.7198	0.6742	0.6787	0.5687
16	0.6613	0.6390	0.5700	0.4688
32	0.4950	0.6464	0.6006	0.5197
64	0.5268	0.4675	0.5115	0.5437
128	0.3867	0.4048	0.4691	0.4861
256	0.4395	0.4617	0.5157	0.3846

**Table 3:** Comparison between Cl-ELISA and Co-ELISA.

Parameters	Cl-ELISA	Co-ELISA
Antigen (mg/L)	5	10
Antibody (mg/L)	0.5	2
Linearity (μg/L)	0.19–25	1.56–200
R^2^	0.9940	0.9893
Reaction time (min)	3.6	10
IC_50_ (μg/L)	1.56	8.15

**Table 4:** Recovery of spiked IMI in vegetable samples (Chinese cabbage, cucumber, and zucchini) using Co-ELISA and Cl-ELISA (n = 3).

	Method 1				Method 1				Method 2			
Sample	Spiked (μg/L)	Co-ELISA (μg/L)	Recovery (%)	RSD (%)	Spiked (μg/L)	Cl-ELISA (μg/L)	Recovery (%)	RSD (%)	Spiked (μg/L)	Cl-ELISA (μg/L)	Recovery (%)	RSD (%)
Chinese cabbage	10	11.35 ± 1.87	113.5	5.4	5	1.34 ± 0.46	26.8	7.5	5	5.47 ± 0.14	109.3	4.7
	50	58.74 ± 1.94	117.5	6.2	10	5.85 ± 1.13	58.5	5.6	10	7.03 ± 0.11	70.3	1.5
	100	81.71 ± 2.02	81.7	6.9	20	18.19 ± 0.56	91.0	1.4	20	13.94 ± 0.17	69.7	8.1
cucumber	10	8.21 ± 1.14	82.1	0.7	5	5.06 ± 0.04	101.2	8.1	5	4.49 ± 0.69	89.7	7.9
	50	49.11 ± 1.20	98.2	1.7	10	7.23 ± 0.09	72.3	24.0	10	11.41 ± 0.47	114.1	1.0
	100	79.38 ± 1.31	79.4	3.2	20	9.30 ± 0.60	46.5	19.5	20	14.82 ± 0.60	74.1	5.5
zucchini	10	11.11 ± 2.05	111.1	7.9	5	9.35 ± 0.30	187.0	43.8	5	6.07 ± 0.17	120.6	5.0
	50	58.81 ± 2.42	117.6	11.1	10	23.74 ± 0.18	237.5	45.7	10	6.99 ± 0.43	69.9	15.0
	100	82.55 ± 2.50	82.5	11.6	20	27.10 ± 0.19	135.5	52.7	20	19.94 ± 0.24	104.9	8.8

**Table 5:** Comparison of instruments detecting IMI (n = 3).

Methods	LOD (μg/L)	Recovery (%)	Sample	RSD (%)	Reference
HPLC—MS/MS	5.9	70.1–119.3	fruits	2.7–12.4	[39]
HPLC	2.7	88.9–93.9	juice	3.2–4.1	[40]
HPLC	10	83.3–90.8	soil	2.4–4.4	[41]
HPLC—MS/MS	2.1	85.9–103.8	vegetable	2.6–12.1	[42]
HPLC	8.53	88.9–90.2	vegetable	5.5–6.8	[43]
ITS	0.04	80.0–124.0	vegetable	0.1–2.7	[36]
Immunoassay (UCNPs/GO)	0.08	76.8–101.8	Water, vegetable, tea, honey	-	[38]
Co-ELISA	1.56	79.4–117.6	vegetable	0.7–11.6	This work
Cl-ELISA	0.19	69.7–120.6	vegetable	1.0–15.0	

ITS: immunochromatographic test strip; reprinted/adapted with permission from Refs. [36,38]. Copyright 2022, Elsevier; reprinted/adapted with permission from Ref. [41]. Copyright 2022, IOP science; reprinted/adapted with permission from Ref. [43]. Copyright 2022, Taylor & Francis; reprinted/adapted with permission from Chinese Refs. [39,40,42].

## Data Availability

The original contributions presented in this study are included in the article, and further inquiries can be directed to the corresponding authors.
